# Brazilian Consumers’ Attitudes towards So-Called “Cell-Based Meat”

**DOI:** 10.3390/foods10112588

**Published:** 2021-10-26

**Authors:** Sghaier Chriki, Vincent Payet, Sérgio Bertelli Pflanzer, Marie-Pierre Ellies-Oury, Jingjing Liu, Élise Hocquette, Jonatã Henrique Rezende-de-Souza, Jean-François Hocquette

**Affiliations:** 1 Isara, AgroSchool for Life, 23 rue Jean Baldassini, 69364 Lyon, France; vpayet@isara.fr (V.P.); elise.hocquette@gmail.com (É.H.); 2 Department of Food Engineering and Technology, University of Campinas, Rua Monteiro Lobato, 80, Campinas 13083-862, SP, Brazil; spflanzer@gmail.com (S.B.P.); jonatarezendesouza@gmail.com (J.H.R.-d.-S.); 3 Bordeaux Science Agro, 33175 Gradignan, France; marie-pierre.ellies@agro-bordeaux.fr; 4 INRAE, Université d’Auvergne, Vetagro Sup, UMR Herbivores, 63122 Saint Genès Champanelle, France; jingjing.liu@inrae.fr (J.L.); jean-francois.hocquette@inrae.fr (J.-F.H.)

**Keywords:** meat alternatives, food security, survey, livestock issues, animal protection, animal welfare, animal production

## Abstract

The main goal of this online survey was to investigate the attitudes of Brazilians towards “cell-based meat”, which has become the subject of great scientific and media enthusiasm. The answers of 4471 respondents concluded that 46.6% of them thought “cell-based meat” was promising and acceptable. More than 66% would be willing to try this novel product compared to 23% who expressed reluctance to do so. Nearly 40% of the total respondents did not want to eat “cell-based meat” regularly at all, whereas 29%, 43.2%, and 39.9% were willing to eat it regularly in restaurants, at home, and/or in ready-made meals, respectively. However, the majority of respondents (71%) were keen to pay much less for “cell-based meat” than conventionally produced meat (or even nothing at all), compared to 24.3% who were willing to pay the same price as conventional meat, whereas only 4.8% were willing to pay more. Approximately 51% of them considered that “cell-based meat” should not be called “meat” for marketing purposes. Job, monthly income, age, and gender were major factors impacting consumer acceptance. Meat professionals and consumers with higher incomes were less willing to eat “cell-based meat” regularly. Women (especially younger women) were the most concerned about the ethical and environmental issues related to meat production and were the most convinced that reducing meat consumption could be a good solution to the meat industry’s problems. Respondents who did not accept “cell-based meat” and did not eat meat substitutes had a negative attitude to this novel food (they considered it absurd and/or disgusting) and did not believe that “cell-based meat” should be called “meat” for marketing purposes. In contrast, the people who thought that “cell-based meat” could be called “meat” perceived it in a rather positive way. These results are important for consumers of meat and meat substitutes and for companies aiming to enter the potential future Brazilian market of “cell-based meat”.

## 1. Introduction

Growing at a rate of approximately 1.05% per year, the global population of 7.8 billion today is expected to surpass 9 billion by 2050. The Food and Agriculture Organization (FAO) has forecast that by 2050, 70% more food will be needed to meet the demands of the growing population [1], which is a major challenge due to limited arable land and water resources [2,3,4]. Furthermore, with the increasing size of the global middle class, developing countries are looking for more and more animal products (meat, cheese, and other dairy products) [5].

In addition, despite the wide range of economic, environmental, cultural, and social services at local, regional, and global levels provided by livestock farming, conventional meat production is facing several challenges, such as environmental and animal welfare issues, climate change, reduced use of antibiotics, food safety, and public health [6].

To address this issue of global food and nutrition security and respond to consumer demand, livestock farming must produce larger quantities of high-quality animal products through production systems that are environmentally sound, socially responsible, and economically viable [1].

However, in this particular context, according to some authors, the most efficient means of protein production are likely to be developed by researchers and start-ups [7,8]. Among these alternatives, “cell-based meat”, also called “cultured meat”, “*in vitro* meat”, or simply “animal proteins” [9], is particularly promoted by its advocates as a sustainable alternative for consumers eager to be more ethically minded but not wishing to change the composition of their diet [10,11,12]. Nevertheless, at this stage, there is no evidence of the sustainability of this alternative protein source, which is still under investigation [13].

The pros and cons of the “cultured meat” process were recently described by Chriki and Hocquette [12]. In this review, the authors updated the current knowledge on this subject by focusing on recent publications and issues that had not been adequately described before.

Initially developed for medical purposes and produced from proliferating cells in a culture medium with hormonal factors [14,15,16], there is no consensus on the health and nutritional qualities of “cell-based meat” for human consumption and its potential low environmental impact [12,17].

The objectives of the proponents of “artificial meat” are to produce high amounts of muscle tissue in order to provide enough meat for the growing human population. After taking a biopsy from a live animal, this piece of muscle is used to release stem cells, which have the ability to proliferate and then differentiate themselves into different types of cells, primarily muscle cells but also fat cells [14].

This novel food is an emerging topic in both the scientific field and news media. Consequently, “cell-based meat” has attracted a lot of media attention, but its presentation varies greatly depending on the media [18], with positive stories of “cultured meat” far outweighing the warnings, according to Painter et al. [19].

Furthermore, a first bibliometric analysis of scientific publications on this subject has highlighted the multidisciplinary nature of the issues related to “cell-based meat” [20]. More recently, a second bibliometric analysis of scientific publications and a sociometric analysis of the mainstream press, conducted by Chriki et al. [9], have identified some potential differences between the scientific view and the public perception of this novel food. It is clear that “cell-based meat” is currently a hot topic in the industrial, political, societal, and scientific spheres, both in the scientific field and in the press media [21].

Although extensive scientific and media coverage has helped to inform the meat industry of this novel food, consumer acceptance has not been sufficiently vetted [22]. Some authors consider that consumer acceptance of the technology is a mandatory first step for the success of this industry [22].

Moreover, some scientists and start-ups working on “cell-based meat” speculate that the COVID-19 pandemic crisis could boost its acceptance and further consumption of this meat alternative, as it would not only decrease the risk of exposure to novel human viral pathogens but also enhance food security and decrease environmental impacts of food production, in line with consumer demand [23,24].

Indeed, consumer acceptance is a complex issue that is currently the subject of investigation by social scientists. A first approach to tackle this problem is to conduct online surveys to examine how consumers will react to “cell-based meat”. Based on the consumers’ perceptions and attitudes towards “cell-based meat”, and their willingness to try, regularly eat, or buy it, these surveys have been conducted in different countries among various consumer groups, mainly focused on Europe [25,26,27,28] and the United States [29], but not exclusively [30,31,32,33,34,35], emphasizing that there were multiple factors influencing acceptance as well as rejection of “cell-based meat”.

In a survey of 1890 scientists and students from all over the world by French authors, Hocquette et al. [36] reported that the majority of respondents considered the technique for the production of “cell-based meat” to be realistic and feasible. However, no majority emerged to think that this novel product would be a good solution from a sensory or nutritional point of view or that it would actually contribute to solving problems of environmental degradation or animal welfare. Only around 10% of the respondents would be willing to consume “cell-based meat” regularly, with the vast majority preferring to reduce their meat consumption, although a third of them were hesitant. Another study conducted in the USA of over 670 people confirmed the high proportion of hesitation and the fact that only a minority (around 10 to 20%) of respondents favored regular consumption of cultured “meat” [29]. A more recent study in Germany confirmed the discrepancy between willingness to “try the product” and willingness to “consume it regularly”, with ultimately a very moderate acceptance of “cell-based meat” [37]. In Italy, consumers would also be willing to try this product at least once, the potential consumer of cultured “meat” being young, highly educated, somewhat familiar with “cell-based meat”, meat-eating, and inclined to reduce consumption [38]. Another survey in Ireland showed that urban consumers were more receptive to “cell-based meat” and more concerned about the environmental impact of “current meat production” practices [39].

However, due to cross-cultural variations in consumer acceptance of “cell-based meat”, the findings from Western countries cannot be fully extrapolated to Brazilian consumers [40]. In addition, it is important to understand the potential attitudes of consumers from countries that are major stakeholders in meat production and consumption. In particular, Brazil is the second largest producer of cattle (after the USA), the fourth largest producer of pigs, and the second largest producer of broiler chickens in the world [41]. Moreover, on average, Brazilians consume nearly 50 kilograms of poultry, more than 25 kilograms of beef and veal, and nearly 13 kilograms of pork per capita each year [42].

However, relatively little research has explored consumer acceptance of “cell-based meat” in Brazil and there is as yet only limited published data with Brazilian participants, with the exception of three available studies conducted before the COVID-19 crisis, and for two of them, by the same research group, each with a limited number of respondents (*n* = 272 [31]; *n* = 626 [43]; *n* = 225 [44]). Therefore, the Brazilian attitude towards “cell-based meat” needs to be explored from several angles. Indeed, more reliable data and new investigations by independent researchers and in the context of the COVID-19 crisis are needed to investigate “cell-based meat” acceptance and the potential demand of Brazilian consumers for this novel food with more confidence that the concept will be fully understood.

The current study therefore seeks to explore the responses of a large group of Brazilian consumers (compared to previous studies) to appropriate questions in order to further investigate their attitudes, perspective, potential acceptance, and willingness to engage with “cell-based meat”, and to provide a broader Brazilian reference for a potential worldwide “cell-based meat” market.

## 2. Methods

This research was approved by the Ethics Committee of the public research university Campinas. commonly known as Unicamp, in the state of São Paulo, Brazil, and has been registered in 2020 under number CAAE: 37924620.5.0000.5404.

### 2.1. Development of the Questionnaire

A Portuguese online questionnaire, entitled “Survey about cell-based meat” was developed on the Google Forms platform (The Portuguese version of the survey is available on: https://cutt.ly/6RULnEV accessed on 13 October 2021).

**Figure 1:**
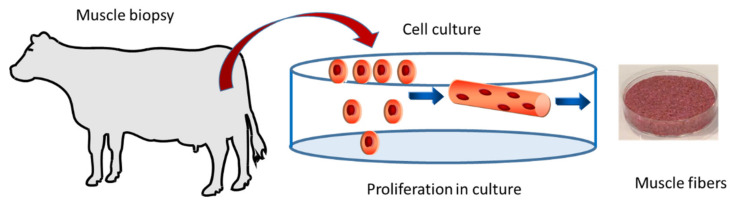
Briefing provided to respondents regarding the introduction of “cell-based meat”.

This 30-question questionnaire was enhanced by an expert in humanities and social sciences and formatted using the Google Forms app. Before starting to answer the questionnaire, an introduction to “cell-based meat” in the form of text (in Portuguese) and a simplified figure of the “cultured meat” process were provided to the respondents (Figure 1). The subsequent survey was composed of a total of 33 questions divided into 6 sections:

(1) Socio-demographic information, (2) Attitude towards societal challenges, (3) Characteristics of the product, (4) Potential interests, (5) Perception, and (6) Development strategies.

After the introduction information, socio-demographic information was collected in the first section, including gender, age, educational level, area of work, net monthly income, meat consumption, and familiarity with “artificial meat”. Two questions were then asked as a preamble regarding respondents’ food purchasing criteria and whether they had ever heard of “cell-based meat”. The second section provided information on respondents’ attitude towards societal challenges facing conventionally produced meat (meat from conventionally raised farm animals) and “cell-based meat”, with regard to ethical, environmental, traditional meat industry, and rural life issues. Next, personal perceptions of this novel food compared to conventionally produced meat were asked in three sections: characteristics of the product, potential interests, and perception. After these three sections, the respondents were asked about the future development strategies to be adopted for the marketing of “cell-based meat”.

### 2.2. Data Collection

The questionnaire was pre-tested on four native-speaking volunteers (graduate students and professors) to identify possible errors of interpretation or inadequate completion. The online survey was conducted in Brazil from 5 August to 5 November 2020, with an estimated duration of 15 minutes per respondent, to investigate the attitude, perspective, acceptance, and willingness to engage with “artificial meat” of Brazilian consumers. The online survey was presented via social media and email in a random sampling method, using a snow-ball sampling method in which respondents were asked to further advertise the survey [43].

The total number of respondents was 4686. For the purpose of this study, in order to understand the behavior of consumers familiar with the Brazilian market, only those who answered Brazil for “origin” (4591) and agreed to save their answers (4471) were kept. Thus, the final sample considered in this study was 4471.

“Cell-based meat”, also known as “artificial meat”, “*in vitro* meat”, “cultured meat”, “lab meat”, “clean meat”, and “synthetic meat”, is a novel food produced in laboratories using animal muscle stem cells, but directly from a living animal, which proliferate in culture. The production of artificial meat is attracting media attention as a way to feed the growing human population. In order to address the increasing concerns about environment (global warming) and ethical issues (animal welfare, animal suffering, and slaughtering) but also the shortcoming of conventionally produced meat production (limited farming resources and ever-increasing population), scientific research is devoted to the introduction and large-scale development of artificial meat as a new meat product in the future.

### 2.3. Statistical Analysis

The data were analyzed using R software [45]. First, the distribution of responses was summarized based on the number and percentage of responses. The responses on a Likert-scale were grouped into five classes: strongly disagree (score 1), mostly disagree (score 2), undecided (score 3), mostly agree (score 4), and strongly agree (score 5).

In order to assess the influence of socio-demographic variables (gender, age, education, job, income, and meat consumption habits) on willingness to try (WTT), pay (WTP), and eat (WTE) “cell-based meat”, these qualitative variables (related to willingness) were coded into quantitative variables:

(i) for WTT: from “Definitely not” = 1 to “Definitely yes” = 5;

(ii) and for WTP: from “Much less” = 1 to “Much more” = 5.

However, for WTE, the question was initially asked with multiple responses and was coded to “0” (do not want to eat “cell-based meat” regularly) and “1” (agree to eat “cell-based meat” regularly). Then, the relationship between socio-demographic information and the variables of willingness (WTT, WTP, and WTE) was performed using ANOVA and completed with a Tukey HSD test (by the R package “agricolae”) for pairwise comparisons between significant groups. Differences were considered significant at a *p*-value < 0.05. The term “data not shown” is cited in this paper when referring to analyses that are not directly presented in tables and figures. This exclusion of these data is particularly related to the length of our paper and the fact that the journal has a figure and table limit.

With such a large amount of data, it is appropriate to use some exploratory techniques to capture the underlying relationship structures between multiple, diverse, and categorical variables. Thus, the relationships between the variables relating to positive drivers, consumer motives and barriers, and willingness regarding the products were studied with a correlation matrix shown in a correlation plot and principal component analysis (PCA). However, for the PCA, only the confidence ellipses for the supplementary qualitative variables were shown. Finally, a partitioning tree for the WTE variable with socio-demographic information (gender, age, education, job, income, meat consumption habits) were computed with the package “rpart”.

## 3. Results

### 3.1. Socio-Demographic Information of Respondents

According to the socio-demographic information detailed in Table 1, the current sample was characterised by a significantly higher number of females (51.5%) than males (48.2%), mainly young and middle-aged (18–50 years old) (77.5%), with a high level of education (graduate and postgraduate, 91.4%). Most of the respondents were not scientists and did not work in the meat sector (68%). Most of them were regular meat-eaters (regularly and daily meat-eaters, 85.9%). Even with a Brazilian minimum wage of 1100 BRL (https://ftp.ibge.gov.br/Trabalho_e_Rendimento/Pesquisa_Nacional_por_Amostra_de_Domicilios_continua/Renda_domiciliar_per_capita/Renda_domiciliar_per_capita_2020.pdf, accessed on 13 October 2021), the respondents had an average income of 3000–10,000 BRL (32.8%, i.e., approximately 552–1840 USD or 454–1513 Euros). In this survey, 73.4% of the respondents were familiar with “cell-based meat” (Table 1).

Moreover, when purchasing food, safety (56.6%), sensorial quality (51.7%), and price (43.7%) were the most important criteria for Brazilian consumers. Environmental impact (6.8%) and nutritional value (3.1%) may not be the priority considerations for most of the current respondents. Ethics, appearance, origin, and labels occupy an intermediate position [26.6–38.1%] in the respondents’ shopping criteria (data not indicated in Table 1).

### 3.2. Effects of Socio-Demographic Factors on Perception, Willingness to Try, Eat, and Pay for “Cell-Based Meat”

#### 3.2.1. Overall Perception

The majority of respondents (46.6%) thought “cell-based meat” was promising and/or acceptable, 35.2% thought this novel food was fun and/or intriguing, and only 18.2% of respondents indicated that “cell-based meat” was absurd and/or disgusting.

#### 3.2.2. Willingness to Try (WTT)

The majority of respondents (66.4%) would be willing to try “cell-based meat” (27.5% definitely yes, 38.9% probably yes), compared to 22.9% who expressed their unwillingness to try this novel product (11.9% definitely not, 11% probably not), and 10.7% were not sure if they would try it (Table A1). However, WTT depends on many factors such as gender, age, job, income, and meat consumption habits as well as the interactions between them (Table A2). In Table 2, only a few very significant pairwise comparisons between demographic groups significant for willingness to try (WTT) are presented.

As shown in Table 2, regardless of gender, young respondents have a higher WTT (>3.80) than older respondents. Young females have the highest WTT (>4) compared to all respondents. However, males over the age of 31 have the lowest WTT (<3.27). Males familiar with the meat sector (meat scientist or worker) have a lower WTT (<2.83) than females and other males who do not know the meat sector. Regardless of gender, respondents with the lowest income (<3000 BRL) have the highest WTT (>3.82), whereas males with the highest income (>15,000 BRL) have the lowest WTT (2.95).

In general, younger respondents (18–30 years) who are not familiar with the meat sector have the highest WTT (>4). However, older (>31 years) meat workers have a lower WTT (<2.94). Overall, young respondents (18–30 years) with low or medium income (<10,000 BRL) have the highest WTT (>3.98), whereas respondents over 51 years of age with the highest income (>15,000 BRL) have the lowest WTT (3.04). Younger respondents (18–30 years) who eat meat rarely and regularly have the highest WTT (>4). Respondents over 31 years of age and who eat meat daily have a lower WTT (3.26) compared to respondents of the same age (>3.66). However, we are unable to observe any obvious relationships between WTT, age, and meat consumption for respondents over 51 years of age.

Scientists who are not familiar with the meat sector with low income (<3000 BRL) have the highest WTT (>4.10), whereas meat workers with a higher income (>10,000 BRL) have the lowest WTT (<2.75). Meat scientist respondents who do not eat meat have the highest WTT (4.83). However, meat workers who eat meat daily have the lowest WTT (2.86), with an intermediate position for the rest of the group (Table 2). Overall, low income respondents (<1500 BRL) eating meat rarely or regularly have the highest WTT (>4.10) (Table 2).

#### 3.2.3. Willingness to Eat Regularly (WTE)

Of the total number of respondents, 39.5% would not eat “cell-based meat” regularly at all. Among the respondents who would eat it regularly, 29%, 43.2%, and 39.9% are willing to eat “cell-based” in restaurants, at home, and/or in ready-made meals (lasagna, hamburger, etc.), respectively (Table A1). However, WTE depends on many factors such as gender, age, education, job, income, and meat consumption habits as well as the interactions between them (except for educational level, Table A3).

As shown in Table 3, among all the respondents, younger females (18–30 years of age) have a higher WTE (0.75) than older males (>31 years of age) who have the lowest WTE (<0.47). Regardless of gender, meat workers and meat scientists have a lower WTE (<0.53) than respondents who do not know the meat sector (WTE > 0.64). Females and males with the lowest income (<3000 BRL) have the highest WTE (>0.75), whereas males with higher income (>7500 BRL) have the lowest WTE (<0.49). Younger respondents (18–30 years) with the lowest income (<1500 BRL) have the highest WTE (0.77), whereas older respondents (>31 years of age) with the highest income (>15,000 BRL) have the lowest WTE (<0.42).

Scientists who are not familiar with the meat sector with low income (<3000 BRL) have the highest WTE (0.82), whereas meat workers with higher income (>10,000 BRL) have the lowest WTE (<0.24). Vegetarian meat scientists, meat workers, and other scientists who rarely eat meat have the highest WTE (>0.86). However, meat workers who eat meat daily have the lowest WTE (0.30), with an intermediate position for the rest of the groups.

Respondents who eat meat regularly with the lowest income (<1500 BRL) have the highest WTE (0.86). Regardless of meat consumption habits, respondents with higher income (>7500 BRL) have the lowest WTE (<0.51) (Table 3).

#### 3.2.4. Willingness to Pay (WTP)

**Figure 2:**
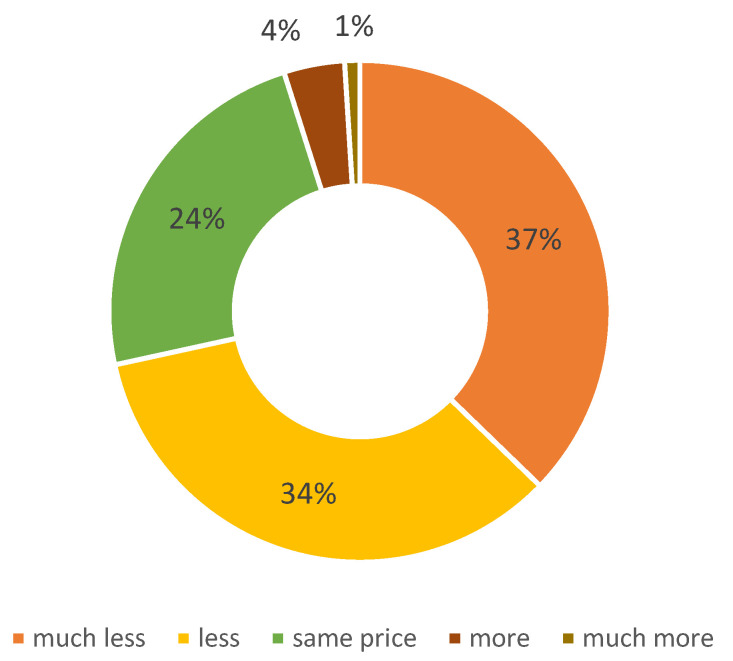
Willingness to pay for “cell-based meat” compared to conventionally produced meat.

The majority of respondents (71%) are willing to pay less or much less for “cell-based meat” than for conventionally produced meat (or even nothing at all), compared to 24% who are willing to pay the same price as conventional meat, whereas only 5% are willing to pay more or much more (Figure 2).

However, this WTP depends on meat consumption and educational level, which also interact (Table A4).

As shown in Table 4, regardless of gender, younger respondents have a higher WTP (>2.07) than older ones. Young females have the highest WTP (2.35) compared to all respondents. However, males over 31 years of age have the lowest WTP (<1.69). In general, regardless of gender, respondents familiar with the meat sector (meat scientists or workers) have the lowest WTP (<1.84). However, females who are not familiar with the meat sector (other scientists or others) have the highest WTP (>2.24). Females with the lowest income (<1500 BRL) have the highest WTP (2.25), whereas males with the highest income (>15,000 BRL) have the lowest WTP (1.55).

Young respondents (18–30 years of age) who are not familiar with the meat sector have the highest WTP (>2.42). However, older respondents (>31 years of age) who work in the meat sector have the lowest WTP (<1.48). Younger respondents (18–30 years of age) with the lowest income (<1500 BRL) have the highest WTP (2.30), whereas respondents over 51 years of age with higher income (1500–7500 BRL) have the lowest WTP (<1.77). Respondents under 50 years of age who do not eat meat have the highest WTP (>2.65) compared to those who eat meat daily (<2.07). Regardless of meat consumption habits, respondents over 51 years of age have a lower WTP (<2.26).

Respondents who are not familiar with the meat sector with less than 15,000 BRL in monthly income have the highest WTP (>2.05), whereas meat workers with the highest income (>15,000 BRL) have the lowest WTP (1.36). Meat scientists who do not eat meat have the highest WTP (4.83). However, meat workers who eat meat daily have the lowest WTP (2.86). Respondents who never eat meat with medium and high incomes (3000–10,000 BRL) have the highest WTP (>2.90), which is the opposite to daily meat-eaters with the highest income (>15,000 BRL) who have the lowest WTP (1.46) (Table 4).

### 3.3. Drivers, Motives, and Barriers of Willingness to Try, Eat Regularly, or Pay for “Cell-Based Meat”

#### 3.3.1. Attitude towards Societal Challenges

The majority (answers with “More (4)” and “Much more (5)”) of respondents agree with the potential ethical (47.7%) and environmental (43.5%) problems related to conventional meat production (Table A5). However, the younger respondents (i.e., under 30 years of age, *n* = 1643), especially the females among them (*n* = 1083), are the most concerned about the ethical and environmental issues related to livestock (data not shown).

Nearly half of the respondents (sum of scores 1 and 2, 46.7%) do not believe that reducing meat consumption could be a good solution to the potential ethical and environmental problems related to conventional meat production (Table A5). However, young females (i.e., under 30 years of age) are the most likely to believe that reducing meat consumption could be a good solution to the potential ethical and environmental problems of meat production, compared to older males (>31 years of age) (data not shown).

In other respects, there is no consensus on the potential interests of “cell-based meat” from an ethical or environmental point of view (Table A5). Similarly, young females believe the most, and older males the least, that “cell-based meat” will be more ethical and environmentally friendly than conventionally produced meat (data not shown).

Additionally, more than 50% of the respondents recognize that “cell-based meat” would have negative impacts on traditional livestock farming and the meat industry, but they are slightly less convinced of its potential negative impact on territories and rural life (35.6% of answers with “More (4)” and “Much more (5)” vs. 41.5% of answers with “Much less (1) and “Less (2)”) (Table A5).

Respondents who worry the most about the potential impacts of “cell-based meat” on traditional livestock farming and on the meat industry are young females, whereas respondents who are the least concerned are males over 51 years of age (data not shown). However, respondents who worry the most about the potential impacts of “cell-based meat” on territories and rural life are males over 31 years of age, compared to females or younger males (>31 years) who worry the least (data not indicated in tables).

#### 3.3.2. Characteristics of the Novel Product

The majority of respondents (60%) believe that “cell-based meat” will be less tasty than conventional meat. In addition, 41.3% consider this novel product to be less healthy, less safe, and of lower nutritional value than traditional meat (Table A6). When results are analyzed by gender and age, young respondents, and especially young females (<30 years of age), believe that “cell-based meat” will be tastier, safer, and have a higher nutritional value than conventional meat (data not indicated in tables).

#### 3.3.3. Perception and Development Strategy

More than half of the respondents (50.6%) have low emotional resistance to trying “cell-based meat” (scores 1 and 2), compared to 32.4% who have high emotional resistance to try it (sum of scores 4 and 5), and 17% of them were unsure (Table A1). Males over 51 years of age show the greatest emotional resistance to trying “cell-based meat” compared to younger respondents (<51 years, regardless of gender) (data not indicated in tables).

The majority of them do not expect “cell-based meat” to be realistic and marketed in the near future (38.9% in the long term (>15 years), 35.6% in the medium term (i.e., from 6 to 15 years), and will never be realistic for 24.8% of respondents), compared to only 12.1% who think it is feasible in the short term (<5 years) (Table A1). In addition, 48.9% of the respondents considered that “cell-based meat” should be called “meat” for marketing purposes, compared to 51.1% who do not agree with this. In the latter case, “Artificial meat” is the term the most cited (31.6%) by the respondents to qualify this novel food product. However, “cellular meat” is the least attractive term (7.8%) according to the respondents (Table A7).

The majority of respondents (sum of scores 1 and 2) answered that both public (62.8%) and private (66.9%) research should invest much more or more resources in “cell-based meat” development. In both cases, between 14.9% and 21.4% were neutral on these aspects (Table A7). In particular, young females (<30 years of age) believe that both public and private research should invest in developing “cell-based meat”, compared to older males (>31 years of age).

#### 3.3.4. Importance of Potential Drivers and Barriers of “Cell-Based Meat” Acceptance

To better understand the positive drivers, motives, and barriers of consumer acceptance of “cell-based meat”, a correlation matrix was performed with all the quantitative variables described above.

**Figure 3:**
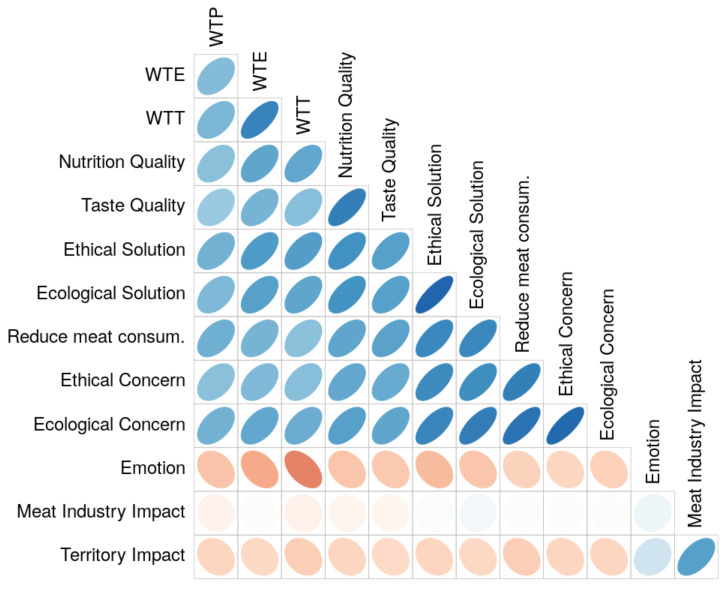
Correlation matrix plot showing relationships between variables. The shapes of the ellipses are opposite for positive (in blue) or negative (in red) correlations. The more intense the color and the thinner the ellipse, the stronger the correlation. The variables are ordered by hierarchical clustering.

As shown in Figure 3, willingness to eat regularly (WTE) and to try (WTT) “cell-based meat” are highly-positively correlated (r = 0.67), which is less true for willingness to pay (WTP) (r < 0.45). Moreover, respondents with high WTE and WTT expressed less emotional resistance (negative correlation in red) to try “cell-based meat”. They also believed that “cell-based meat” will be more ethical and environmentally friendly than conventional meat, and that both private and public research should invest more in developing this novel food. However, the respondents who were the most concerned about the ethical and environmental issues related to livestock believed that “cell-based meat” will be more ethical and environmentally friendly than conventional meat. Conversely, respondents with the lowest WTE and WTT are the most concerned about the potential negative impact of “cell-based meat” on territories, rural life, and the meat industry.

#### 3.3.5. Comparison between “Cell-Based Meat” and Meat Alternatives

Regardless of their acceptance of “cell-based meat”, a large proportion of respondents (71.3%) do not consume meat substitutes. Approximately 55% of them will accept “cell-based meat” as a viable alternative to conventional meat in the future, compared to other meat substitutes (such as soy protein) or other meat alternatives (such as reducing food waste or developing our farming practices). However, 45.1% of respondents (regardless of whether they already eat meat substitutes) do not accept this novel food as a good alternative to conventional meat.

As detailed previously, PCA was performed on the variables related to the positive drivers, motives, and barriers of consumers and the willingness regarding the product. The first axis (horizontal) explains 47% of the variability of all the responses, with high scores for all the variables, except for three of them (territorial and industrial impacts of “cell-based meat”, and emotional resistance to try “cell-based meat”). Moreover, the territorial and industrial impacts of “cell-based meat” are linked to the second axis (vertical).

**Figure 4:**
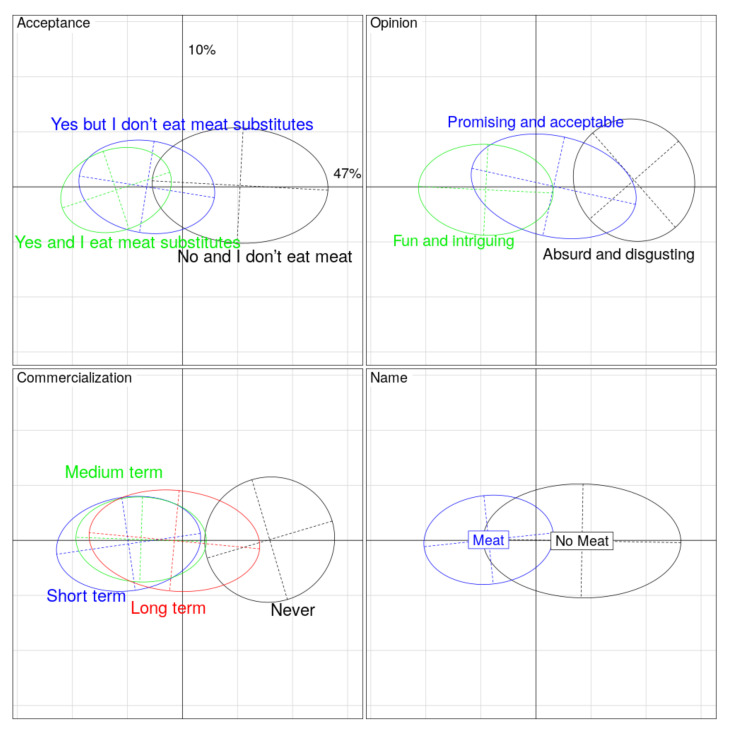
Principal component analyses showing the relationships between variables. In each graph are shown the first axis (horizontal, which explains 47% of the variance) and the second one (vertical, which explains 10% of the variance). We show the confidence ellipses for four qualitative variables: “Acceptance”, “Opinion”, “Commercialization/Marketing”, and “Name”.

Regardless of whether they already eat meat substitutes or not, the respondents who accept “cell-based meat” as a good alternative to conventional meat thought that “cell-based meat” is promising and/or acceptable, and feasible in the short term (<5 years). Moreover, they considered that “cell-based meat” should be called “meat” for marketing purposes. Conversely, when respondents perceived “cell-based meat” as “absurd and/or disgusting”, the same negative attitudes can be seen with the other components that are “I do not accept “cell-based meat” as a meat alternative”, “cell-based meat will never be marketed” and “cell-based meat” should not be called as “meat” (Figure 4).

**Figure 5:**
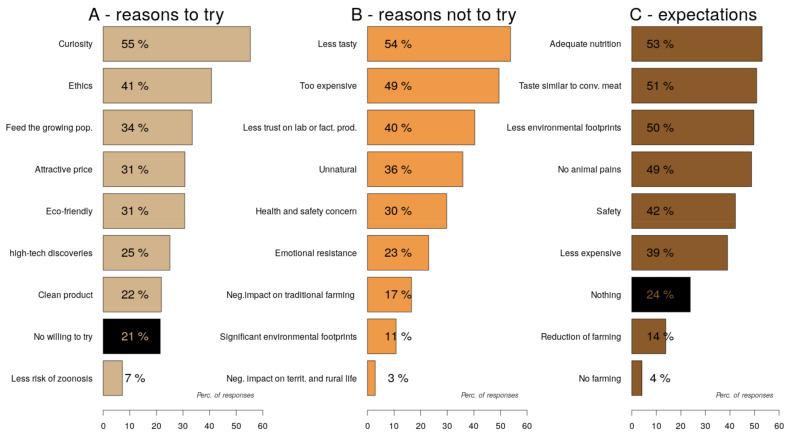
Reasons for engaging (**A**), obstacles (**B**), and expectations (**C**) of “cell-based meat” (multiple choice questions).

All these elements of interpretation are confirmed by two other questions of the survey. Overall, although 78.5% of respondents agreed to try “cell-based meat” at least once (but 21.5% had no willingness to try it), to the question “Why would you be willing to try cell-based meat?”, curiosity (55.3%) and ethics (40.7%) are presented as the most important reasons to engage with “cell-based meat”. The food crisis, attractive price, environmental reasons, and attraction to high-tech discoveries are presented as the second most important reasons (21.9–33.5%) associated with willingness to try this novel product. Prevention of zoonosis is not the main concern (7.2%) of the respondents (Figure 5A).

On the contrary, to the question “Why would you not be willing to try cell-based meat?”, sensory (53.8%) and economic (49.5%) aspects were frequently chosen as the main reasons for no willing to try whereas environmental footprints and negative impact on territories and traditional farming were the lowest obstacles to engaging with “cell-based meat”. Unnaturalness, lack of safety, and lab product phobia occupy intermediate positions (Figure 5B).

In terms of expectations of “cell-based meat”, adequate nutrition, taste, safety, low carbon footprints, and less animal suffering were the most frequently selected options (48.8–53.1%) compared to other options such as safety, lower price, or no farming. Nearly 24% of respondents had no expectations of “cell-based meat” (Figure 5C).

### 3.4. Hierarchical Classifications of Quantitative and Qualitative Variables and Respondents

**Figure 6:**
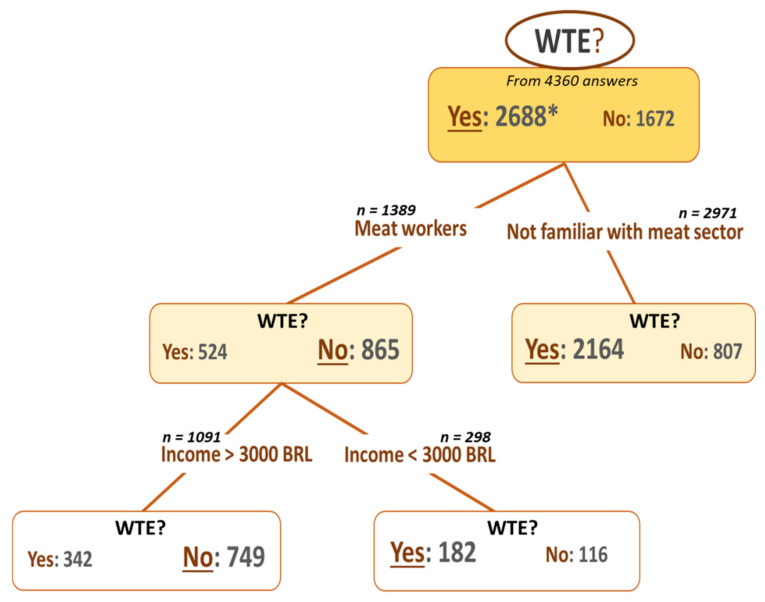
Partition tree of willingness to eat “cell-based meat” regularly (WTE) according to socio-demographic information (gender, age, education, job, and meat consumption habits). * The number of “Yes” or “No” responses to the eating “cell-based meat” willingness to eat regularly question is given in front of the text “Yes” or “No”. The most chosen response among all the respondents is entered in the box with a larger font (“Yes” for the first box, for example).

Firstly, as shown in Figure 6, professional activity explains the choice of the respondents to eat “cell-based meat” regularly or not. Indeed, meat workers are less willing to eat this novel food regularly (number of “No” = 865). In contrast, the respondents who are not familiar with the meat sector are recognized as the largest group of people who would be willing to eat “cell-based meat” regularly (number of “Yes” = 2164). Second, among meat workers, respondents earning more than 3000 BRL per month are less willing to eat “cell-based meat” regularly (number of “No” = 749), unlike respondents with lower incomes (<3000 BRL; number of “Yes” = 182) (Figure 6).

## 4. Discussion

### 4.1. What Type of Consumer Would Be Willing to Engage with “Cell-Based Meat” in Brazil?

#### 4.1.1. “Cell-Based Meat” Is More Attractive to Young People (18–30 Years of Age) in Brazil

Age and gender are major factors that impact people’s food choices [46]. In agreement with this is that age is one of the most important effects in this study. Indeed, younger respondents have the highest willingness to consume (WTT and WTE) “cell-based meat” compared to older respondents (>31 years of age). In addition, the younger respondents have, at the same time, a higher WTP compared to older respondents. This result should be interpreted with caution, because among all young respondents, the majority (59%) are willing to pay less or much less for “cell-based meat” than conventional meat (or even nothing at all), compared to 34% who are willing to pay the same price as for conventional meat, and only 7% of them are willing to pay more.

Moreover, younger respondents are more concerned about the ethical and environmental issues related to animal husbandry, and tend to think of “cell-based meat” in terms of their own consumption, whereas older people tend to think of it in terms of an implicit societal transition (for review: [47]). This is not the case for older Chinese respondents (>51 years of age) who are more willing to engage with “cell-based meat”, unlike younger people [35].

Nonetheless, our results are in accordance with the Brazilian study by Heidemann et al. [31] who argued that young people may be more concerned about animal welfare issues, leading to a higher willingness to try alternative proteins [48]. The fact that older people reject “cell-based meat” to a greater extent confirms the results of another survey in Germany and France [49] and a recent European survey [46], both of which show that older respondents (65 years of age or older) have low acceptance of “cell-based meat”. In contrast, in an Australian survey on the Generation Z, (18–24 years of age), considered to be familiar with technologies and with a sense of environmental responsibility, Bogueva and Marinova [30] reported that 72% of respondents from this younger generation are not ready to accept “cell-based meat” (compared to 3.1% of younger people between 18 to 30 years of age in our study who find this idea absurd and/or disgusting). The way in which the questions are worded or the difference in age or maturity of the respondents may explain this type of different observation.

#### 4.1.2. “Cell-Based Meat” Is More Attractive to Women in Brazil

Gender influences concerns about the ethical and environmental problems caused by the meat industry, opinions on meat consumption, and overall perception of “cell-based meat” [29].

In our study, women are the most concerned about the ethical and environmental issues related to livestock and believe the most that reducing meat consumption could be a good solution to the problems of the meat industry. Our results confirm those of Heidemann et al. [31] who indicate that women specialists in animal production are more in favour (65%) of “cell-based meat” than men. This could be related to women’s sensitivity to animal welfare and environmental issues related to farming and that they are more likely to follow different diets including the flexitarian diet (with less meat consumption), according to Ruby and Heine [50]. However, in another recent survey [49], it was observed that women are less inclined to consume this novel food than men, in Germany and especially in France. Likewise, Wilks and Phillips [29] concluded that men are more willing to engage with “cell-based meat” than women.

This type of contradiction can be explained by an interaction between age and gender, as it was observed that younger women and older men have the most similar perceptions [36]. However, despite a significant interaction between age and gender, our study shows, on the contrary, that older men are more reluctant. The different wording of the questions between the surveys could explain at least in part these contradictions.

#### 4.1.3. “Cell-Based Meat” Is More Attractive to People with Low Income in Brazil

In our study, respondents with the lowest monthly income (<3000 BRL) have the highest acceptance (WTT and WTE) of “cell-based meat” than respondents with the highest income (>15,000 BRL).

This is consistent with the study by Wilks and Phillips [29] who concluded that respondents with low income are more willing to engage with “cell-based meat” than participants with higher incomes who viewed this meat alternative as less ethical, according to these authors. This is not in accordance with other studies emphasizing that meat alternatives were generally more appealing to higher income groups, which means that the balance of data is unclear on this point [47]. Even with significantly higher values for WTP in lower income classes, for some classes of respondents, it is observed that in general the results of WTP were low, close to, or below 2.0, which shows that consumers would not be willing to pay more for the product compared to conventional meat (Table 4). However, in our study, respondents with a low income (<3000 BRL) also have a higher WTP compared to richer respondents (>15,000 BRL per month). With a Brazilian minimum wage around 1100 BRL per month in 2021, this higher WTP should be analyzed with caution as among respondents with low income (<3000 BRL), almost 60% are willing to pay less or much less for “cell-based meat” than conventional meat (or even nothing at all), compared to 34% who are willing to pay the same price as conventional meat, and only 6% of them are willing to pay more.

Furthermore, there is an interaction between age and income among all young respondents, as the younger respondents (18–30 years of age) with the lowest income have the highest WTP, whereas respondents over 51 years of age with higher income have the lowest WTP. It is likely that respondents with low income hope to have access to a cheapest protein food in the future, whereas richer respondents are not willing to pay for this novel food product, despite earning more money per month. In contrast, in the study of Liu et al. [35], Chinese young people, who are beginners in being economically independent, being still students, may tend to be more conservative (i.e., with lowest WTT, WTE, and WTP) towards new products, especially if the price of the new product is too high.

### 4.2. Area of Work Is a Relevant Predictor of Willingness to Eat “Cell-Based Meat” Regularly

According to Heidemann et al. [31], the opinion of professionals involved in animal production is very important for the development of the emerging “cell-based meat” chain and its future potential development.

In our study, the majority of meat professionals are firmly opposed to “cell-based meat”, with the lowest acceptance (WTT, WTE, and WTP) of “cell-based meat” compared to respondents who are not familiar with meat sector. These results confirm those of Heidemann et al. [31] who indicated that animal scientists have a lot of reservations and resistance to “cell-based meat”, associating this biotechnology with “unnaturalness”, which has a negative connotation. Moreover, they assume that they do not have job opportunities in the “cell-based meat” industry, and believe, consequently, that “cell-based meat” would be a danger to their respective and current jobs, if it were to one day replace “conventional meat”.

This is in contradiction with the results of Bryant et al. [49] who argued that working in the agricultural sector is a factor favorable to the acceptance of “cell-based meat.” However, our results confirm other previous findings that showed that the urban population (less familiar with livestock and conventional meat production) would be more willing to consume “cell-based meat” [51,52]. Indeed, a survey conducted in Ireland showed that urban consumers were more receptive to “cell-based meat” and more concerned about the environmental impact of “current meat production” practices [39]. Rural consumers were more concerned about the potentially negative effects that “cell-based meat” production could have on agriculture and the farmers’ lifestyles [39]. This result is confirmed by another study showing that the potential consumer of “cell-based meat” is described as young, highly educated, somewhat familiar with this novel food, a meat-eater, and inclined to reduce consumption [38].

### 4.3. The Brazilian Consumer’s Perspective Is of Importance for the Acceptance of “Cell-Based Meat” as a Viable Alternative to Conventional Meat

Respondents who do not accept “cell-based meat” and do not consume meat substitutes have a negative attitude towards this novel food (absurd and/or disgusting) and, more importantly, they do not believe that “cell-based meat” should be called “meat” after it has been marketed. This is in line with another survey in which the majority of respondents stated that cultured meat is not “natural” and considered it a technological product rather than meat [53]. This is also aligned with the point of view of the American Meat Science Association that this novel product does not meet yet the definition of meat [54].

In contrast, the people who think that “cell-based meat” can be called “meat” perceive it in a rather positive way. This confirms the importance of the perception of this novel product as “meat” or not in the potential acceptance and development of this product [9,55]. It has been shown that wordings can positively affect consumer acceptance [56]. For example, consumers with the perception of a “high tech” product are less likely to buy or eat this product, in contrast to consumers exposed to terms that emphasize the societal advantages of “cell-based meat” (such as “clean meat”) or similarity to “conventional meat” [9,55]. Thus, the use of the word “meat” has induced an ambiguity favorable to the advocates of cultured “meat” who seek to do away with the negative values associated with meat (environmental degradation and animal suffering), while taking advantage of the positive values of meat in the minds of consumers (strength, vitality, healthiness, etc.) [9].

However, in general, the majority of respondents are willing to pay much less or less for “cell-based meat” than for conventional meat as previously observed with Chinese respondents [35] even if the proportion is higher in China (87% vs. 71%). A major challenge of the “cultured-meat” industry is thus to design a cheap product to enter the mass market in both countries.

### 4.4. Opinion Surveys Should Be Interpreted with Caution Due to Their Limited Representative Nature

The representativeness of those surveyed in our study in relation to the Brazilian population should be put into perspective. Compared to the Brazilian national data from 2019 [57], our participants were slightly younger and had a slightly higher income and education level (Table 1). However, these data are still broadly representative of the population and should therefore provide insight into general Brazilian attitudes.

Regarding the gender distribution, in 2019 it is balanced in the Brazilian population with 51.8% women vs. 48.2% men, not very different from our sample (51.5% women).

In addition, in the Brazilian population, there are 32.9% of people aged over 51 years (under-represented in our sample with only 22.5%), 33.7% of citizens between 31 and 50 years of age (over-represented in our sample with 40.8%), and 20.2% of young people between 18 to 30 years of age (also over-represented with 36.7% in our sample). These differences reflect a greater interest of young people in science, thus inducing a greater willingness on their part to answer this type of questionnaire. This type of bias in surveys has already been observed, for example, in the survey by Heidemann et al. [31]. However, these drawbacks can be partially offset by the consequent size of the sample (*n* = 4471), making it possible to analyze each segment of the population, our survey being the one with the largest number of people in the population to our knowledge.

According to the OECD [58], Brazil had only 0.2% of citizens with a PhD and 0.8% a master’s degree. As for the other diplomas, IBGE [57] accounted for 16.5% with a bachelor’s degree, 76.1% with short vocational studies, and 6.4% with no previous education.

However, as in the study by Oliveira et al. [44], the respondents to our survey were more highly educated (graduates and postgraduates, 91.4%), which is explained by the fact that the questionnaire was initially distributed through scientific networks (universities, research institutes, etc.). As students are more interested in science than others, one can assume that they respond more easily to this kind of survey. In addition, in the Brazil population, there are 0.1% of scientists (almost 200 thousand; [59], against 24.8% in our survey, which is easily explained by the interest of scientists in innovations as well as our mainly university dissemination network). This is in agreement with the conclusions of Heidemann et al. [31] who indicated that biotechnology engineers would be more likely to accept “cell-based meat” based on their knowledge of the field as well as the professional opportunities generated by this technology after it has been marketed.

In Brazil, 8.2% of citizens work directly in the agriculture sector and 1.4% in the meat sector (production and industry) [60], whereas this sector of activity represents 32% in our study sample. The results must therefore be corrected for this type of factor. In our study, 32.8% of respondents have an average income (3000–10,000 BRL, i.e., around 552–1840 USD or 454–1513 Euros) compared to approximately 20.0% of Brazilians who have an average income above 3650 BRL [57].

The results can also be potentially affected by the respondents’ diet. In Brazil, 8% of Brazilians declared themselves vegetarians in 2018 [61]. In our study, vegetarians and vegans represent only 6.7%, which is not different from the study by Heidemann et al. [31], in which vegetarians and vegans represent 8.1% of respondents.

Finally, more generally, the formulation and sequencing of the questions can change the interpretation and therefore the results [47]. Furthermore, although the survey was conducted in the most neutral way possible, the choice of questions and their order can also influence the people surveyed in one direction or another. Indeed, some studies are carried out by promoters of “cell-based meat” with the explicit objective of convincing consumers [62] or accelerating the marketing of “cell-based meat” [47,49]. Finally, due to the importance of the implicit attitudes as described above, a recent survey illustrated the insufficiency of relying on self-reported measures when seeking to capture consumers’ opinions of unfamiliar or unknown products such as “cell-based meat” [63]. However, despite these limitations, comparing results obtained with the same experimental design between countries (such as China as published by Liu et al. [35], France, and Brazil) or between similar social groups (within the same study as in this work) is likely to provide useful information for understanding the motives and barriers to “cultured meat” acceptance.

## 5. Conclusions

This study investigated consumers’ attitudes, perspectives, willingness and potential acceptance of “cell-based meat” in Brazil, one of the major stakeholders in the production and consumption of meat in the world. Although opinion surveys should be interpreted with caution due to their limited representativeness from a socio-demographic point of view, with a high number of respondents, this study provides novel results on the potential acceptance of “cell-based meat” in Brazil, as it was also conducted by a group of researchers belonging to public research organizations and not linked to private companies seeking to market this biotechnology, unlike some previous surveys. Age and gender are major factors impacting consumer acceptance. Although they would not be willing to pay more for “cell-based meat” (if it is ever marketed) than for conventional meat, younger respondents have the highest willingness to consume “cell-based meat” compared to older respondents (>31 years of age). Furthermore, qualified as more sensitive to animal husbandry issues, women (especially the youngest among them) are more concerned about the ethical and environmental issues related to meat production and believe the most that reducing meat consumption could be a good solution to the problems of the meat industry. This is why, as previously shown in other surveys, reducing the consumption of meat (known as called “flexitarianism”) is preferred, at the expense of “cell-based meat” consumption, which is not necessarily considered more ethical or eco-friendly than conventional meat, by Brazilian respondents. Finally, meat professionals and people with higher incomes are less willing to eat “cell-based meat” regularly.

These results are important for consumers of meat and meat substitutes and for companies aiming to enter the Brazilian market to boost their development. For example, because consumers are not willing to pay more for this novel product than for conventional meat, the price of “cell-based meat” should drop significantly, and become even cheaper than conventional meat. The potential future market for “cellular meat” in Brazil should also consider the growing trend of modern eating habits based on convenient snacking.

## 6. Highlights

- The answers of 4471 respondents concluded that 46.6% of them thought “cell-based meat” was promising and acceptable.
- More than 66% were willing to try “cell-based meat”, whereas 71% were willing to pay much less than conventional meat (or even nothing at all).
- Nearly 40% of the total respondents did not want to eat “cell-based meat”.
- Consumers with different socio-demographic characteristics (job, monthly income, age, and gender) showed different attitudes to “cell-based meat”.
- Approximately 51% of them considered that “cell-based meat” should not be called “meat” for marketing purposes.

## Figures and Tables

**Table 1:** Socio-demographic information of respondents.

Question	Response Option	*n* ^a^	% ^b^
Gender	Female	2303	51.5
	Male	2156	48.2
	Does not wish to answer	12	0.3
Age	18–30 years of age	1643	36.7
	31–50 years of age	1825	40.8
	>51 years of age	1003	22.5
Education	Primary and middle school	265	6.0
	Graduate	1957	43.7
	Postgraduate degree	2130	47.6
	Other (PhD, Master, Technician, etc.)	92	2.1
	Does not wish to answer	27	0.6
Area of work	Meat scientist	327	7.3
	Other scientist	782	17.5
	Meat worker	1104	24.7
	Others	2258	50.5
Net monthly income	<1500 BRL ^c^	524	11.7
	1500–3000 BRL	696	15.6
	3000–7500 BRL	1004	22.5
	7500–10,000 BRL	460	10.3
	10,000–15,000 BRL	559	12.5
	>15,000 BRL	759	17.0
	Does not wish to answer	469	10.4
Meat consumption	Never: vegetarian or vegan diet	299	6.7
	Rarely: weekly or less	331	7.4
	Regularly: several times a week	1739	38.9
	Daily or at each meal	2102	47.0
Familiarity ^d^	Yes	3282	73.4
	No	1189	26.6

^a^*n*: number of responses; ^b^ %: percentage of responses (total = 4471); ^c^ BRL: Brazilian Real, 1 BRL = 0.18 USD or 0.15 Euro; ^d^ familiarity: Have you ever heard of artificial meat?

**Table 2:** Pairwise comparisons between significant demographic groups for willingness to try (WTT) “cell-based meat”.

	**< Age >**			**< Area of Work >**				
	**18–30**	**31–50**	**>51**	**Meat S ^1^**	**Meat W ^2^**	**Other** **S ^3^**	**Others ^4^**	
**Gender**								
Female	4.01 ^a^	3.73 ^b^	3.49 ^c^	3.72 ^ab^	3.39 ^bc^	4.03 ^a^	3.85 ^a^	
Male	3.80 ^b^	3.27 ^d^	3.07 ^d^	3.27 ^c^	2.83 ^d^	3.90 ^a^	3.63 ^b^	
**Income** ^**5**^								
	**<1.5 k**	**1.5–3 k**	**3–7.5 k**		**7.5–10 k**	**10–15 k**	**>15 k**	
**Gender**								
Female	3.93 ^a^	3.92 ^a^	3.77 ^a^		3.93 ^a^	3.76 ^ab^	3.54 ^abc^	
Male	3.87 ^a^	3.82 ^a^	3.37 ^c^		3.38 ^bc^	3.26 ^c^	2.95 ^d^	
**< Area of Work >**					**< Meat Consumption >**			
	**Meat S ^2^**	**Meat W ^3^**	**Other S ^4^**	**Others ^5^**	**Never**	**Rarely**	**Regularly**	**Daily**
**Age**								
18–30	3.63 ^bc^	3.40 ^c^	4.16 ^a^	4.09 ^a^	3.66 ^bc^	4.40 ^a^	4.13 ^a^	3.81 ^b^
31–50	3.57 ^c^	2.94 ^d^	3.98 ^ab^	3.63 ^c^	3.72 ^bc^	3.89 ^ab^	3.66 ^bc^	3.26 ^c^
>51	3.09 ^cd^	2.56 ^d^	3.54 ^c^	3.46 ^c^	2.79 ^d^	3.60 ^bc^	3.43 ^c^	2.87 ^d^
**Meat Consumption**								
		**Never**	**Rarely**		**Regularly**		**Daily**	
**Area of work**								
Meat S		4.83 ^a^	4.29 ^ab^		3.59 ^ab^		3.44 ^ab^	
Meat W		3.46 ^ab^	4.00 ^ab^		3.16 ^ab^		2.86 ^b^	
Other S		3.44 ^ab^	4.12 ^ab^		4.14 ^ab^		3.89 ^ab^	
Others		3.58 ^ab^	3.88 ^ab^		3.84 ^ab^		3.69 ^ab^	
**Income**								
		**<1.5 k**	**1.5–3 k**	**3–7.5 k**	**7.5–10 k**	**10–15 k**	**>15 k**	
**Area of work**								
Meat S		3.89 ^abc^	3.84 ^abc^	3.09 ^cde^	3.51 ^abcde^	3.50 ^bcde^	3.26 ^cde^	
Meat W		3.53 ^abcd^	3.50 ^bcde^	3.03 ^de^	2.99 ^de^	2.75 ^e^	2.69 ^e^	
Other S		4.0 ^ab^	4.10 ^a^	4.0 ^ab^	3.97 ^ab^	4.05 ^ab^	3.62 ^abc^	
Others		3.98 ^ab^	3.93 ^ab^	3.78 ^abc^	3.82 ^abc^	3.63 ^abc^	3.31 ^cde^	
**Meat Consumption**								
**Income**		**Never**	**Rarely**		**Regularly**		**Daily**	
<1.5 k		3.35 ^b^	4.23 ^a^		4.10 ^a^		2.90 ^b^	
1.5–3 k		3.51 ^ab^	4.02 ^ab^		3.94 ^ab^		3.87 ^ab^	
3–7.5 k		3.85 ^ab^	3.81 ^ab^		3.76 ^ab^		3.37 ^b^	
7.5–10 k		4.09 ^ab^	4.06 ^ab^		3.83 ^ab^		3.35 ^b^	
10–15 k		3.38 ^ab^	3.83 ^ab^		3.72 ^ab^		3.23 ^b^	
>15 k		3.13 ^b^	4.03 ^ab^		3.28 ^b^		3.86 ^ab^	

^1^ Meat S: meat scientist; ^2^ Meat W: meat worker; ^3^ Other S: scientist working outside the meat sector; ^4^ Others: not scientist and outside the meat sector; ^5^ k: 1000, e.g., 1.5 k = 1500; ^a–e^: different letters in the same section indicate means with significant statistical differences at the 0.05 level. Scale: 1 to 5 (from “Definitely not” = 1 to “Definitely yes” = 5).

**Table 3:** Pairwise comparisons between significant demographic groups for willingness to eat “cell-based meat” regularly.

	**< Age >**			**< Area of Work >**			
	**18–30**	**31–50**	**>51**	**Meat S ^1^**	**Meat W ^2^**	**Other S ^3^**	**Others ^4^**
**Gender**							
Female	0.75 ^a^	0.69 ^b^	0.62 ^b^	0.53 ^bc^	0.48 ^c^	0.79 ^a^	0.75 ^a^
Male	0.66 ^b^	0.47 ^c^	0.43 ^c^	0.41 ^cd^	0.29 ^d^	0.74 ^a^	0.64 ^b^
**Income** ^**5**^							
	**<1.5 k**	**1.5–3 k**	**3–7.5 k**		**7.5–10 k**	**10–15 k**	**>15 k**
**Gender**							
Female	0.76 ^a^	0.75 ^a^	0.68 ^ab^		0.75 ^a^	0.67 ^abc^	0.57 ^bcd^
Male	0.75 ^a^	0.71 ^ab^	0.55 ^cd^		0.49 ^d^	0.48 ^d^	0.36 ^e^
**Income**							
	**<1.5 k**	**1.5–3 k**	**3–7.5 k**		**7.5–10 k**	**10–15 k**	**>15 k**
**Age**							
18–30	0.77 ^a^	0.75 ^ab^	0.65 ^bcd^		0.70 ^abcd^	0.71 ^abcd^	0.58 ^cde^
31–50	0.74 ^abc^	0.72 ^abc^	0.65 ^bcd^		0.61 ^cde^	0.53 ^cde^	0.39 ^e^
>51	0.64 ^bcde^	0.70 ^abcd^	0.53 ^cde^		0.58 ^cde^	0.58 ^cde^	0.42 ^de^
**Meat Consumption**							
	**Never**		**Rarely**		**Regularly**	**Daily**	
**Area of work**							
Meat S	1.00 ^a^		0.85 ^ab^		0.47 ^ab^	0.46 ^ab^	
Meat W	0.58 ^ab^		0.86 ^a^		0.38 ^ab^	0.30 ^b^	
Other S	0.55 ^ab^		0.87 ^a^		0.82 ^ab^	0.74 ^ab^	
Others	0.64 ^ab^		0.73 ^ab^		0.73 ^ab^	0.69 ^ab^	
**Income**							
	**<1.5 k**	**1.5–3 k**	**3–7.5 k**		**7.5–10 k**	**10–15 k**	**>15 k**
**Area of work**							
Meat S	0.80 ^ab^	0.54 ^bcde^	0.35 ^def^		0.51 ^bcdef^	0.29 ^ef^	0.38 ^def^
Meat W	0.63 ^abc^	0.54 ^bcd^	0.40 ^def^		0.32 ^ef^	0.24 ^f^	0.22 ^f^
Other S	0.79 ^ab^	0.82 ^a^	0.79 ^ab^		0.76 ^ab^	0.75 ^ab^	0.70 ^ab^
Others	0.78 ^ab^	0.79 ^ab^	0.71 ^ab^		0.70 ^ab^	0.72 ^ab^	0.50 ^cdef^
**Meat Consumption**							
	**Never**		**Rarely**		**Regularly**	**Daily**	
**Income**							
<1.5	0.50 ^bc^		0.83 ^ab^		0.86 ^a^	0.74 ^abc^	
1.5–3 k	0.57 ^bc^		0.78 ^abc^		0.78 ^abc^	0.72 ^abc^	
3–7.5 k	0.73 ^abc^		0.78 ^abc^		0.67 ^bc^	0.55 ^bc^	
7.5–10 k	0.81 ^abc^		0.78 ^abc^		0.67 ^bc^	0.51 ^c^	
10–15 k	0.65 ^bc^		0.70 ^abc^		0.66 ^bc^	0.45 ^c^	
>15 k	0.45 ^c^		0.68 ^abc^		0.43 ^c^	0.38 ^c^	

^1^ Meat S: meat scientist; ^2^ Meat W: meat worker; ^3^ Other S: scientist working outside the meat sector; ^4^ Others: not scientist and outside the meat sector; ^5^ k: 1000, e.g., 1.5 k = 1500; ^a–f^: different letters in the same section indicate means with significant statistical differences at the 0.05 level. Scale: “0” (do not want to eat “cell-based meat” regularly) and “1” (agree to eat “cell-based meat” regularly).

**Table 4:** Pairwise comparisons between significant demographic groups for willingness to pay for “cell-based meat”.

	**< Age >**				**< Area of Work >**				
	**18–30**	**31–50**	**>51**		**Meat S ^1^**	**Meat W ^2^**		**Other S ^3^**	**Others ^4^**
**Gender**									
Female	2.35 ^a^	2.06 ^b^	1.95 ^b^		1.84 ^c^	1.80 ^c^		2.36 ^a^	2.24 ^ab^
Male	2.07 ^b^	1.69 ^c^	1.62 ^c^		1.74 ^c^	1.42 ^d^		2.16 ^b^	1.95 ^c^
**Income** ^**5**^									
	**<1.5 k**	**1.5–3 k**		**3–7.5 k**	**7.5 k–10 k**			**10–15 k**	**>15 k**
**Gender**									
Female	2.25 ^a^	2.15 ^ab^		2.16 ^ab^	2.23 ^ab^			2.17 ^ab^	2.03 ^ab^
Male	2.22 ^ab^	1.96 ^bc^		1.75 ^c^	1.73 ^cd^			1.75 ^c^	1.55 ^d^
	**< Area of Work >**				**< Meat Consumption >**				
	**Meat S**	**Meat W**	**Other S**	**Others**	**Never**		**Rarely**	**Regularly**	**Daily**
**Age**									
18–30	1.93 ^cd^	1.72 ^d^	2.46 ^a^	2.42 ^a^	2.76 ^a^		2.61 ^ab^	2.32 ^bc^	2.07 ^d^
31–50	1.77 ^cd^	1.48 ^e^	2.21 ^b^	1.99 ^c^	2.65 ^a^		2.25 ^bcd^	1.94 ^d^	1.67 ^e^
>51	1.49 ^de^	1.41 ^e^	2.04 ^bc^	1.86 ^cd^	2.26 ^bcd^		2.12 ^cd^	1.77 ^de^	1.57 ^e^
**Income**									
	**<1.5 k**	**1.5–3 k**		**3–7.5 k**	**7.5 k–10 k**			**10–15 k**	**>15 k**
**Age**									
18–30	2.30 ^a^	2.24 ^ab^		2.17 ^abc^	2.38 ^a^			2.18 ^abc^	2.27 ^ab^
31–50	1.95 ^abc^	1.85 ^bc^		1.93 ^bc^	1.93 ^bc^			1.89 ^bc^	1.62 ^c^
>51	1.86 ^bc^	1.69 ^c^		1.77 ^c^	1.83 ^bc^			1.92 ^bc^	1.65 ^c^
**Meat Consumption**									
	**Never**			**Rarely**			**Regularly**	**Daily**	
**Area of work**									
Meat S	4.83 ^a^			4.29 ^ab^			3.59 ^ab^	3.44 ^ab^	
Meat W	3.46 ^ab^			4.00 ^ab^			3.16 ^ab^	2.86 ^b^	
Other S	3.44 ^ab^			4.12 ^ab^			4.14 ^ab^	3.89 ^ab^	
Others	3.58 ^ab^			3.88 ^ab^			3.84 ^ab^	3.69 ^ab^	
**Income**									
	**<1.5 k**	**1.5–3 k**		**3–7.5 k**	**7.5–10 k**			**10–15 k**	**>15 k**
**Area of work**									
Meat S	1.93 ^bcdef^	1.94 ^bcde^		1.75 ^cdefg^	1.90 ^bcdefg^			1.50 ^efg^	1.72 ^cdefg^
Meat W	1.93 ^bcdef^	1.66 ^defg^		1.52 ^efg^	1.40 ^fg^			1.56 ^efg^	1.36 ^g^
Other S	2.39 ^ab^	2.34 ^ab^		2.40 ^a^	2.22 ^abc^			2.27 ^ab^	2.03 ^bcd^
Others	2.32 ^ab^	2.15 ^abc^		2.10 ^abc^	2.12 ^abc^			2.05 ^bc^	1.85 ^cdefg^
**Meat Consumption**									
	**Never**			**Rarely**			**Regularly**	**Daily**	
**Income**									
<1.5 k	2.54 ^abc^			2.52 ^abc^			2.27 ^bcd^	2.10 ^cdef^	
1.5–3 k	2.26 ^bcde^			2.24 ^bcde^			2.10 ^cdef^	2.02 ^cdef^	
3–7.5 k	2.90 ^a^			2.38 ^abcd^			1.93 ^defg^	1.82 ^efg^	
7.5–10 k	3.27 ^a^			2.38 ^abcd^			2.02 ^cdef^	1.75 ^fg^	
10–15 k	2.90 ^ab^			2.22 ^bcdef^			1.94 ^defg^	1.80 ^efg^	
>15 k	2.65 ^abc^			2.00 ^cdefg^			1.84 ^efg^	1.46 ^g^	

^1^ Meat S: meat scientist; ^2^ Meat W: meat worker; ^3^ Other S: scientist working outside the meat sector; ^4^ Others: not scientist and outside the meat sector; ^5^ k: 1000, e.g., 1.5 k = 1500; ^a–g^: different letters in the same section indicate means with significant statistical differences at the 0.05 level. Scale: 1 to 5 (from “Much less” = 1 to “Much more” = 5).

## Data Availability

The data presented in this study are available on request from the corresponding author. The data are not publicly available because they concern consumers’ expression.

## Appendix A

**Table A1:** Responses about willingness to engage with cell-based meat.

Question	Response Option	*n* ^a^	% ^b^
Emotional resistance (e.g., disgusting, weird) to try cell-based meat	1 Much less	1669	37.9
	2 Less	568	12.7
	3 Neutral/Unsure	758	17
	4 More	553	12.4
	5 Much more	896	20
Willingness to try cell-based meat	Definitely not	533	11.9
	Probably not	492	11
	Neutral/Unsure	478	10.7
	Probably yes	1737	38.9
	Definitely yes	1231	27.5
Context and willingness to eat cell-based meat regularly	At the restaurant	1296	29
	At home	1932	43.2
	In ready-to-eat meals: lasagnes, hamburger…	1785	39.9
	I do not want to eat cell-based meat regularly	1764	39.5
Future commercialization of cell-based meat	On the short term: from 1 to 5 years	542	12.1
	On the medium term: from 5 to 15 years	1591	35.6
	On the long term: more than 15 years	1739	38.9
	Never	1111	24.8

^a^*n*: number of responses; ^b^ %: percentage of responses (total = 4471).

**Table A2:** Analysis of variance of willingness to try cell-based meat according to demographics.

Willingness to Try	Df	F Value	*p*
Gender	1	150.7	<0.001 ***
Age	2	106.7	<0.001 ***
Education	8	1.35	0.2138
Area of work	3	123.3	<0.001 ***
Income	5	36.0	<0.001 ***
Meat Consumption	3	29.2	<0.001 ***
Gender × Age	2	4.3	0.01312 *
Gender × Education	8	1.4	0.1727
Gender × Area of work	3	5.2	0.00126 **
Gender × Income	5	4.3	0.00062 ***
Gender × Meat consumption	3	1.8	0.1445
Age × Education	16	2.7	0.0002 ***
Age × Area of work	6	2.5	0.0188 *
Age × Income	10	1.8	0.04245 *
Age × Meat consumption	6	2.9	0.0075 **
Education × Area of work	20	1.6	0.034 *
Education × Income	30	1.4	0.05857
Education × Meat consumption	21	1.3	0.147
Area of work × Income	15	1.9	0.017 *
Area of work × Meat consumption	9	3.1	0.0009 ***
Income × Meat consumption	15	2.7	0.000364 ***

Significance codes: ‘***’ *p* < 0.001 ‘**’ *p* < 0.01 ‘*’ *p* < 0.05; either *p* > 0.05.

**Table A3:** Analysis of variance of willingness to eat cell-based meat regularly according to demographics.

Willingness to Eat	Df	F Value	*p*
Gender	1	190.8	<0.001 ***
Age	2	73.6	<0.001 ***
Education	8	2.5	0.00871 **
Area of work	3	198.6	<0.001 ***
Income	5	46.2	<0.001 ***
Meat Consumption	3	31.3	<0.001 ***
Gender × Age	2	6.9	0.00097 ***
Gender × Education	8	0.6514	0.73471
Gender × Area of work	3	3.5	0.01393 *
Gender × Income	5	5.7	<0.001 ***
Gender × Meat consumption	3	2.3	0.0717
Age × Education	16	1.5	0.06337
Age × Area of work	6	1.1	0.34230
Age × Income	10	1.6	0.08558
Age × Meat consumption	6	2.3	0.02743 *
Education × Area of work	20	1.5	0.05707
Education × Income	30	1.2	0.20095
Education × Meat consumption	21	1.1	0.27035
Area of work × Income	15	3.9	<0.001 ***
Area of work × Meat consumption	9	5.0	<0.001 ***
Income × Meat consumption	15	3.4	<0.001 ***

Significance codes: ‘***’ *p* < 0.001 ‘**’ *p* < 0.01 ‘*’ *p* < 0.05; either *p* > 0.05.

**Table A4:** Analysis of variance of willingness to pay for cell-based meat according to demographics.

Willingness to Pay	Df	F Value	*p*
Gender	1	150.7	<0.001 ***
Age	2	106.7	<0.001 ***
Education	8	1.35	0.2138
Area of work	3	123.3	<0.001 ***
Income	5	28.4	<0.001 ***
Meat Consumption	3	29.2	<0.001 ***
Gender × Age	2	4.3	0.01312 *
Gender × Education	8	1.4	0.1727
Gender × Area of work	3	5.2	0.00126 **
Gender × Income	5	5.5	<0.001 ***
Gender × Meat consumption	3	1.8	0.1445
Age × Education	16	2.7	0.0002 ***
Age × Area of work	6	2.5	0.0188 *
Age × Income	10	1.2	0.2323
Age × Meat consumption	6	2.9	0.0075 **
Education × Area of work	20	1.6	0.034 *
Education × Income	30	0.9	0.53143
Education × Meat consumption	21	1.3	0.147
Area of work × Income	15	1.2	0.1943
Area of work × Meat consumption	9	3.1	0.0009 ***
Income × Meat consumption	15	4.2	<0.001 ***

Significance codes: ‘***’ *p* < 0.001 ‘**’ *p* < 0.01 ‘*’ *p* < 0.05; either *p* > 0.05.

**Table A5:** Responses about societal challenges caused by conventionally produced meat and cell-based meat.

Question	Response (1: Much Less—5: Much More)				
	1 ^a^	2 ^b^	3 ^c^	4 ^d^	5 ^e^
Do you think the conventionally produced meat farming and industry cause ethical problems (e.g., animal suffering, slaughtering…)?	918 ^6^ (20.5) ^7^	565 (12.6)	855 (19.1)	771 (17.2)	1362 (30.5)
Do you think the conventionally produced meat farming and industry cause environmental problems (e.g., huge water consumption, greenhouse gas emissions...)?	915 (20.5)	499 (11.2)	668 (14.9)	701 (15.7)	1688 (37.8)
Do you think reducing meat consumption could be a good solution to resolve above problems?	1603 (35.9)	485 (10.8)	688 (15.4)	507 (11.3)	1188 (26.6)
How ethical do you think cell-based meat would be compared to conventionally produced meat?	997 (22.3)	512 (11.5)	1038 (23.2)	847 (18.9)	1077 (24.1)
How eco-friendly do you think cell-based meat would be compared to conventionally produced meat?	984 (22)	526 (11.8)	1044 (23.4)	920 (20.6)	997 (22.3)
Do you think cell-based meat have negative impacts on traditional livestock farming and meat industry (e.g., employment…)?	578 (12.9)	535 (12)	974 (21.8)	1036 (23.2)	1348 (30.1)
Do you think cell-based meat have negative impacts on territories and rural life (e.g., biodiversity, tourism, landscape maintenance, vitality of the territories…)?	1026 (22.9)	832 (18.6)	1019 (22.8)	725 (16.2)	869 (19.4)

^a^ 1 Much less, ^b^ 2 Less, much less-1 and less-2 sometimes were analyzed together; ^c^ 3 Neutral/Unsure; ^d^ 4 More, ^e^ 5 Much more, more-4 and much more-5 sometimes were analyzed together; ^6^ Number of responses; ^7^ Percentage of responses (total = 4471).

**Table A6:** Responses about characteristics of the future cell-based meat product.

Question	Response (1: Much Less—5: Much More)				
	1 ^a^	2 ^b^	3 ^c^	4 ^d^	5 ^e^
How healthy, safe and with a high-nutritional-value do you think cell-based meat will be compared to conventionally produced meat (i.e., in terms of proteins, vitamins…)?	1065 ^6^ (23.8) ^7^	784 (17.5)	1509 (33.8	686 (15.3)	427 (9.6)
How tasty do you think cell-based meat will be compared to conventionally produced meat (i.e., look like conventionally produced meat in terms of taste and appearance)?	1633 (36.5)	1050 (23.5)	1113 (24.9)	408 (9.1)	267 (6)

^a^ 1 Much less, ^b^ 2 Less, much less-1 and less-2 sometimes were analyzed together; ^c^ 3 Neutral/Unsure; ^d^ 4 More, ^e^ 5 Much more, more-4 and much more-5 sometimes were analyzed together; ^6^ Number of responses; ^7^ Percentage of responses (total = 4471).

**Table A7:** Responses about the future strategy to develop cell-based meat.

Question	Response Option	*n* ^a^	% ^b^
Which name is the most attractive for you to qualify artificial meat?	Artificial meat	1410	31.6
	In vitro meat	606	13.6
	Clean meat	813	18.2
	Cultured meat	1088	24.3
	Cellular meat	350	7.8
	Lab meat	979	21.9
	Synthetic meat	1211	27.1
	Animal-free meat	588	13.2
	Slaughter-free meat	1123	25.1
To which extend do you think that scientific public research must mobilize means (time and funding) in this biotechnology?	1 Much less	692	15.5
	2 Less	301	6.7
	3 Neutral/Unsure	668	14.9
	4 More	829	18.5
	5 Much more	1981	44.3
To which extend do you think that the private research model (by start-ups) is relevant for possibly developing research on artificial meat?	1 Much less	344	7.7
	2 Less	184	4.1
	3 Neutral/Unsure	955	21.4
	4 More	1022	22.9
	5 Much more	1966	44
If this product is commercialized one day, do you think it should be named “meat”?	Yes	2185	48.9
	No	2286	51.1

^a^*n*: number of responses; ^b^ %: percentage of responses (total = 4471).
